# Single-cell foundation models: bringing artificial intelligence into cell biology

**DOI:** 10.1038/s12276-025-01547-5

**Published:** 2025-10-01

**Authors:** Seungbyn Baek, Kyungwoo Song, Insuk Lee

**Affiliations:** 1 Department of Biotechnology, College of Life Science & Biotechnology, Yonsei University, Seoul, Republic of Korea; 2 Department of Statistics and Data Science, Yonsei University, Seoul, Republic of Korea; 3 DECODE BIOME Co., Ltd., Incheon, Republic of Korea

**Keywords:** Computational models, Machine learning

## Abstract

A foundation model, a large-scale deep learning model pretrained on vast datasets, has revolutionized data interpretation through self-supervised learning with capacity for various downstream tasks. Concurrently, single-cell genomics is in urgent need of unified frameworks capable of integrating and comprehensively analyzing the rapidly expanding data repositories. Inspired by advances in foundation models, researchers have extended these techniques to single-cell analysis, giving rise to single-cell foundation models (scFMs). Typically, these models use transformer architectures to incorporate diverse omics data and extract latent patterns at both cell and gene/feature levels for the analysis of cellular heterogeneity and complex regulatory networks. Despite their promise, scFMs face challenges including the nonsequential nature of omics data, inconsistency in data quality and the computational intensity required for training and fine-tuning. Furthermore, interpreting the biological relevance of latent embeddings and model representations remains nontrivial. Here we provide an overview of scFMs, highlighting their key concepts and applications across downstream tasks. We critically assess the current limitations and propose future directions aimed at enhancing the robustness, interpretability and scalability of scFMs. Ultimately, addressing these challenges will be crucial for establishing scFMs as pivotal tools in advancing single-cell genomics and unlocking deeper insights into cellular function and disease mechanisms.

## Transforming single-cell genomics with foundation models

In recent years, scientists have gathered a lot of data from individual cells, which help us understand how different cells work in various conditions. Researchers are now using artificial intelligence models, called foundation models, to make sense of these data. These models can learn from large datasets and be adapted for specific tasks. Foundation models are trained on vast amounts of data to recognize patterns. In single-cell biology, these models treat each cell like a sentence and genes like words. This approach helps the model understand the ‘language’ of cells. Researchers use these models to predict cell types, correct data errors and even simulate how cells might react to changes. Results show that these models can perform many tasks well, but they still face challenges such as handling rare cell types and ensuring data quality.

This summary was initially drafted using artificial intelligence, then revised and fact-checked by the author.

## Introduction

Since the advent of high-throughput single-cell sequencing, vast collections of single-cell data have become available across diverse tissues and conditions. Numerous atlas studies profiling cells from various tissues and multiple species exemplify the scale and diversity of data now accessible. Although many single-cell studies address targeted biological questions, there is a growing interest in unified models that can leverage these large, heterogeneous datasets. In parallel, transformer-based architectures have revolutionized natural language processing (NLP) and computer vision by capturing intricate long-range relationships in data. These architectures enable the development of foundation models defined as large-scale, self-supervised artificial intelligence (AI) models trained on diverse datasets that can be adapted for a wide range of downstream tasks^1–3^. Inspired by successes in text and image domains, researchers have begun developing single-cell foundation models (scFMs) that learn from extensive single-cell datasets and can be fine-tuned for various biological analyses. In this Review, we summarize the key concepts underlying scFMs and discuss their applications in downstream single-cell data analysis. We also highlight the current limitations of these models and suggest future directions for improving their performance and utility in biological research.

## What are foundation models?

Foundation models are large AI models that are trained on extensive datasets at scale and then adapted to a wide range of tasks. A defining feature is their training via self-supervised objectives often through predicting masked segments, enabling the model to learn generalizable patterns. These models develop rich internal representations that can be fine-tuned to excel in specific tasks with relatively few additional labeled examples. Originally popularized in natural language^4^ and vision domains^5^, foundation models learn a bedrock of knowledge that can support diverse applications. Key components contributing to their success include: (1) training on extremely large and diverse datasets to capture universal patterns to be utilized for various general tasks, (2) effective architectures often based on transformer that can model complex dependencies and (3) the ability to fine-tune or prompt the model for new tasks, transferring the learned knowledge to improve performance on target tasks. In several cases, foundation models also leverage data augmentation or synthetic data generation to enrich the training corpus, and they may implicitly model the probability distribution of the data, allowing them to generate new samples^6^.

## Concept, architecture and development of scFMs

### scFM as a large language model for single-cell biology

With the accumulation of large-scale single-cell datasets in the public domain, single-cell biology now provides fertile ground needed for foundation models. The public domain contains tens of millions of single-cell omics datasets, spanning many cell types, states and conditions. Using this wealth of data, researchers have started to train large models to decipher the ‘language’ of cells. In these scFMs, individual cells are treated analogously to sentences, and genes or other genomic features along with their values are treated as words or tokens^7^. The premise is that by exposing a model to millions of cells encompassing many tissues and conditions, the model can learn the fundamental principles of cells and their features that are generalizable to new datasets or downstream tasks.

Most scFMs so far have focused on single-cell RNA sequencing (scRNA-seq) data, learning from gene expression matrices. However, several models have capacities to incorporate additional modalities such as single-cell ATAC sequencing (scATAC-seq)^8,9^, multiome sequencing^7^, spatial sequencing^10,11^ and single-cell proteomics^12^ to create more comprehensive foundation models. Early attempts at scFMs appeared around 2022, with transformer models such as single-cell bidirectional encoder representations from transformers (scBERT)^13^ trained on millions of single-cell transcriptomes in a self-supervised manner for cell type annotation. Since then, several large-scale scFMs have been introduced, each leveraging massive single-cell corpora^7,11,14–19^. Although these models vary in architecture and training details, they share the goal of learning a unified representation of single-cell data that can drive many downstream analyses.

### Data sources for pretraining

**Fig. 1:**
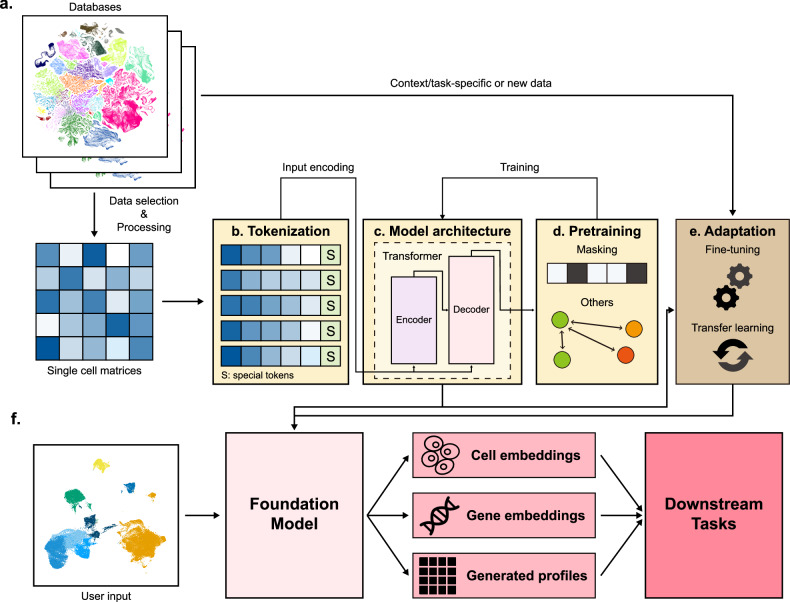
Overview of scFMs. **a** Single-cell datasets are aggregated from public databases, repositories and manual searches. These datasets are processed to serve as pretraining data for scFMs, with a subset or new dataset selected to update the models to specific contexts or conditions. The example uniform manifold approximation and projection (UMAP) plots are from The Allen Brain Cell Atlas. **b**, Each cell in the pretraining datasets is converted into a model input by tokenizing its gene expressions or other measured profiles, with special tokens optionally added to denote particular cell characteristics. **c** The model architecture is built using multiple deep neural network layers, typically arranged in the transformer architecture. **d** The pretraining is carried out using various pretraining tasks, such as masking strategies and contrastive learning, among others. **e** After the initial pretraining, the model can be fine-tuned and further adapted via continual pretraining or transfer learning to meet specialized purposes or contexts. **f** The user inputs are fed into the scFMs to extract cell embeddings, gene embeddings and generated feature profiles, which can be utilized for a variety of downstream tasks.

A critical ingredient for any foundation model is the compilation of large and diverse datasets (Fig. 1a). For scFMs, researchers benefit from archives and databases that have organized vast amounts of publicly available data sources. Platforms such as CZ CELLxGENE provide unified access to annotated single-cell datasets, with over 100 million unique cells standardized for analysis^20^. Likewise, the Human Cell Atlas and other multiorgan atlases provide a broad coverage of cell types and states^21^. Public repositories such as the National Center for Biotechnology Information (NCBI) Gene Expression Omnibus (GEO) and Sequence Read Archive (SRA) and the EMBL-EBI Expression Atlas host thousands of single-cell sequencing studies. Integrating these studies yields extensive training corpora. Curated compendia such as PanglaoDB^22^ and the Human Ensemble Cell Atlas^23^ collate data from multiple sources and studies.

These aggregated data enable scFMs to be trained on cells with various biological conditions ideally capturing a wide spectrum of biological variation. However, challenges in data quality arise owing to different experiments having varying depth, batch effects, technical noise and varying processing steps^24–26^. Although there is no single best practice for data selection and processing yet, effective pretraining requires the careful selection of datasets, filtering of cells and genes, balances in dataset compositions and quality controls^17,18,24,27,28^. Overall, assembling a high-quality, nonredundant dataset for pretraining is as important as model architecture in building a robust scFM.

### Tokenization

Tokenization refers to the process of converting raw input data into a sequence of discrete units called tokens. Tokenization is necessary because it standardizes raw, often unstructured data into structured data that models can understand, process and learn. In NLP, these tokens are often words or subwords in sentences. In scFMs, tokenization involves defining what constitutes a ‘token’ from single-cell data, typically representing each gene (or feature) as a token. These tokens serve as the fundamental input units for the model, analogous to words in a sentence. One of the most important considerations for a successful generation of scFM is a method for input representation or tokenization. For most models, genes or features become input tokens, and the combinations of these tokens collectively represent a single cell (Fig. 1b). However, additional approaches are often required to successfully generate input for the model. One fundamental challenge is that gene expression data are not naturally sequential. Unlike words in a sentence, genes in a cell have no inherent ordering. To apply transformers, input genes generally have an order or structure. A common strategy is to rank genes within each cell by their expression levels and then feed the ordered list of top genes as the ‘sentence’^11,14,17,29^. This provides a deterministic but arbitrary sequence based on expression magnitude. Other models partition genes into bins by their expression values and use those rankings to determine their positions^7,13,15^. However, several models report no clear advantages for complex ranking strategies and simply use normalized counts^19^. Each gene is typically represented as a token embedding that might combine a gene identifier and its expression value in the given cell. With the various strategies above, positional encoding schemes are adapted to represent the relative order or rank of each gene in the cell.

Additional special tokens may be inserted to enrich the input. For example, several models prepend a token representing the cell’s own identity and metadata, enabling the model to learn cell-level context^7,19,30^. If multiple omics are used, tokens indicating modality can be included^7,11^. Gene metadata such as gene ontology or chromosome location can also be incorporated to provide more biological context^16,19,31^. Whereas several models report robustness to batch-dependent technical biases without incorporating batch-specific tokens^14,28^, other models incorporate batch information as special tokens^7^. After tokenization, all tokens are converted to embedding vectors, which are then processed by the transformer layers. The result of the forward pass is typically a set of latent embeddings: one for each gene token and often a dedicated embedding for the entire cell. These input embeddings are fed into a model to be used for pretraining tasks for model training.

### Model architecture

Most of the well-known and recently successful foundation models in NLP and vision are built on the transformer architecture, which has likewise become the backbone of scFMs. Transformers are neural network architectures characterized by attention mechanisms that allow the model to learn and weight the relationships between any pair of input tokens^32,33^. In large language models, this allows the model to decide which words in a sentence to focus on when predicting the next word or a masked word. By analogy, in scFMs, the attention mechanism can learn which genes in a cell are most informative of the cell’s identity or state, how they covary across cells and how they have regulatory or functional connections. The gene expression profile of each cell is converted to a set of gene tokens, serving as inputs for the model, and its attention layers gradually build up a latent representation of each cell or gene.

Most scFMs use some variant of the transformer with different architectural configurations (Fig. 1c). Several adopt a bidirectional encoder representations from transformers (BERT)-like encoder architecture with bidirectional attention mechanism where the model learns from the context of all genes in a cell simultaneously^13^. Others, such as the single-cell generative pretrained transformer (scGPT), use an architecture inspired by the decoder of the Generative Pretrained Transformer (GPT), with a unidirectional masked self-attention mechanism that iteratively predicts masked genes conditioned on known genes^7^. There are also models with an encoder–decoder combination or custom modifications^34^. Whereas these architectures are known to have certain strengths (that is, encoder model for classification and embedding and decoder model for generation) in the broader foundation model landscape^35,36^, such characteristics have not been clearly tested or compared against scFMs. So far, no single architecture has emerged as clearly superior for single-cell data—both encoder-based and decoder-based scFMs have shown success, and hybrid designs are being explored.

### Pretraining strategies

Pretraining an scFM means training it on a self-supervised task across the unlabeled single-cell data so that it learns generalizable patterns. Most scFMs incorporate certain forms of gene prediction tasks, often with masking for pretraining depending on their model architecture (Fig. 1d). Masked language modeling is commonly used with encoder-based models. A random subset of genes in each cell’s token list is masked, and the model is trained to predict these missing genes on the basis of the context from the other genes in the cell. By learning to infer masked genes, the model must capture coexpression relationships and global structure in gene expression profiles^37^. Autoregressive (next-token) modeling is used with decoder-based models. This strategy involves ordering the genes in a cell and training the model to predict the next gene token in the sequence. The model learns from a prefix of genes to predict which gene comes next, thus modeling a distribution over gene expression patterns^37^. scGPT followed this approach but enhanced with a specialized attention-masking mechanism to define the order of prediction^7^.

In addition to these primary language-inspired objectives, several scFMs incorporate auxiliary pretraining tasks to inject more biological structure. For models with special metadata tokens, the models might be tasked with distinguishing or predicting a cell’s label^19^. Furthermore, the gene prediction tasks could be replaced with a task to reconstruct numeric expression values or ranks of genes rather than just identities^19,38^. These multitask pretraining schemes can guide the model to encode features that are especially useful for specific applications. Likewise, contrastive learning between cell pairs has been used to ensure that cells with similar profiles end up with similar embeddings, improving the model’s ability to separate cell types^15^. The computational resources for pretraining scFMs are highly variable. Models with tens to hundreds of millions of parameters typically require several days to a few weeks on a small cluster of modern graphics processing units (GPUs)^7,13,14,18,19^ (for example, 4-12 NVIDIA A100s/H100s), whereas bigger models with more extensive parameters or datasets can necessitate larger clusters for over a month^16^. In summary, pretraining strategies for scFMs generally start with large language-modeling objectives and may extend with additional objectives to imbue the model with biologically relevant inductive biases.

### Model adaptation

After initial pretraining, models can be further updated to adapt to specific conditions, downstream tasks, or new datasets (Fig. 1e). Several large models undergo continual pretraining, where the model is first pretrained on a base corpus and then incrementally updated with new data or new tasks^18^. This can be useful to incorporate emerging single-cell datasets or to adapt a general model to a specific context.

One of the greatest advantages of foundation models is their ability to be fine-tuned. After initial pretraining, a model can be further trained on a specific task or dataset to generate a specialized model, rather than training a new model from scratch for each task. Fine-tuning allows scFMs to achieve high performance on downstream tasks by leveraging the knowledge acquired during pretraining and updating the model for certain downstream tasks often with task-specific datasets^2,7^. The task-specific datasets for fine-tuning can vary dramatically in size, from hundreds to millions of cells, contingent upon the task and available data^7,13,14^. These datasets might be curated subsets of the pretraining corpus, frequently with supplementary information, or they may introduce novel contexts, such as perturbation effects or tumor environments, enabling the model to generalize to previously unseen conditions.

Commonly, several layers of the transformer are frozen during fine-tuning and only certain layers are updated. This prevents overfitting and preserves the general knowledge while adapting the model to the new task. Fine-tuning has been shown to substantially boost the performance of scFMs^2^. For these reasons, many models incorporate or recommend fine-tuning for certain downstream tasks.

Given the large size of scFMs, model inference and fine-tuning can be computationally expensive, particularly for researchers with limited access to large computing clusters, and several models are beginning to explore parameter-efficient techniques^18^. One promising approach is low-rank adaptation (LoRA), which adds a small number of trainable parameters to each transformer attention layer instead of updating the full weight matrices, drastically reducing the resources needed for fine-tuning^39^. This has been used in NLP and could be applied to scFMs to update the models for new tasks with minimal changes. Another approach is model quantization, which involves reducing the numerical precision of model weights to shrink the model size and memory usage^40^. The task-specific nature of fine-tuning, coupled with parameter-efficient techniques, generally makes it less resource-intensive than pretraining. However, more extensive updates or training on larger fine-tuning datasets can still be computationally demanding.

In summary, the fine-tuning step allows a single pretrained scFM to be adapted for many applications. Each fine-tuned model shares the same backbone but is slightly tweaked for optimal performance on the given problems or datasets. This versatility is a key reason why foundation models are appealing in single-cell biology. They are generalist models that can become experts in many different tasks, as needed.

### Model deployment for downstream tasks

To perform multiple downstream tasks, scFMs incorporate user-provided datasets into either pretrained or fine-tuned models to extract cell and gene/feature embeddings and attention weights or to generate feature profiles (Fig. 1f). Initially, matrices of cell by gene by output embedding dimensions from the last or penultimate transformer layers are extracted and subsequently processed for downstream analysis.

**Fig. 2:**
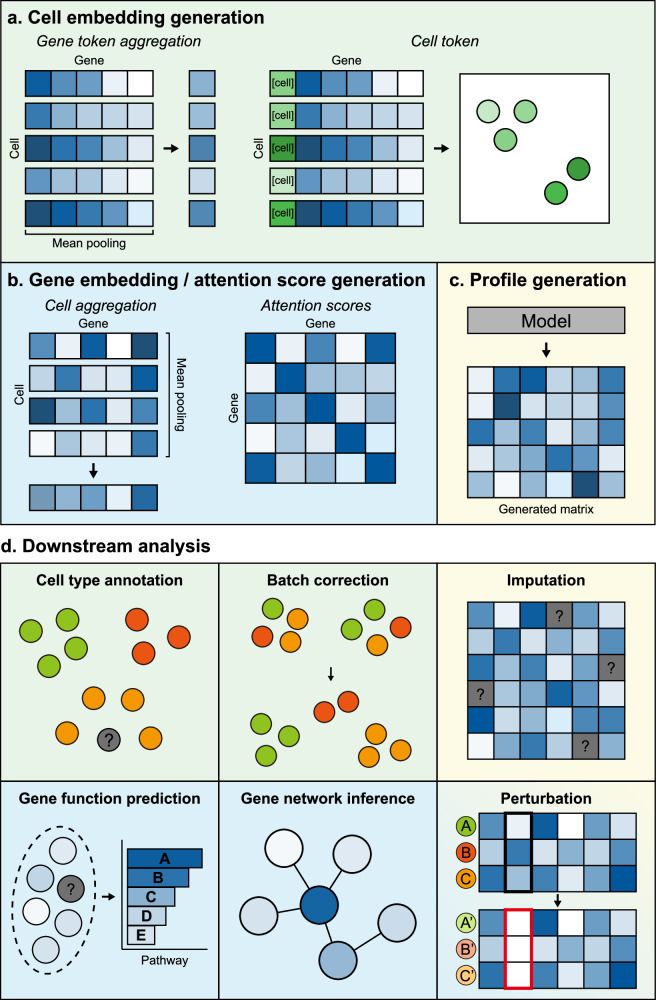
Model outputs and downstream tasks. **a** The cell embeddings are derived either by aggregating gene tokens within each cell or by appending and training special tokens (cell tokens, often classification tokens) that represent individual cells. **b** The gene embeddings are produced by pooling gene-specific embeddings from all input cells. In addition, attention scores from selected layers and heads capture the relationships among genes. **c** The models generate feature profiles that represent the distribution of cells across different conditions or states. **a**–**c** explain initial outputs generated by feeding inputs to scFMs. **d** Various downstream tasks with scFMs. Each task is colored with types of model-generated output utilized for the task.

Cell embeddings capture the relative positioning of cells within latent spaces generated by deep neural layers from foundation model architectures. These embeddings are typically generated by aggregating gene embeddings for each cell through mean or max pooling. Additionally, cell embeddings can be directly represented by the special tokens (often called classification tokens), which are trained along with other input tokens (Fig. 2a).

For gene/feature-level downstream tasks, scFMs leverage gene embeddings or attention scores that reflect gene-to-gene relationships. For a given group of cells, each gene is represented by aggregated gene embeddings from the cells. Moreover, the attention scores are extracted from selected attention heads and model layers (Fig. 2b).

For downstream tasks that require generation or prediction of feature profiles, the models are often fine-tuned to reflect specific conditions. The desired feature profiles are extracted and further compared or processed (Fig. 2c).

## Applications of scFMs in downstream analysis

scFMs are transforming the analysis of single-cell data by providing comprehensive, multipurpose frameworks for a wide range of downstream tasks including cell type annotation, batch correction and integration, imputation, gene function prediction, gene network generation/inference and perturbation prediction and in silico generation (Fig. 2d). These models leverage deep learning to generate gene/feature and cell embeddings as well as generated feature profiles that can be used for these tasks. A key strength of scFMs lies in their versatility. They can be readily adapted for multiple tasks, often demonstrating competitive performance even without task-specific fine-tuning or design (zero-shot mode). Despite their promise, benchmarking the performance of scFMs remains challenging (see the ‘Benchmarking and evaluation’ section in Current limitations and challenges). Still, several reports have highlighted the distinct strengths for certain downstream tasks such as cell type prediction, gene function prediction and spatial imputation even in the zero-shot setting, while also identifying weaknesses and areas needing improvement such as expression imputation, cross-platform batch integration and gene network inference^2,41,42^. Importantly, fine-tuning scFMs on task-specific datasets generally leads to substantial gains in performance and enables more complex applications that may not be feasible in zero-shot settings^2,41^. As these models and benchmarking frameworks continue to develop, there remains substantial room for improvement in both accuracy and generalizability. With the ongoing expansion of pretraining datasets and methodological refinements, scFMs hold substantial potential to advance our understanding of cellular heterogeneity and regulatory mechanisms.

### Cell type annotation

The accurate cell type identification is a critical initial step in single-cell analysis. Traditional approaches rely on canonical marker genes and clustering, which can be subjective or labor-intensive^43^. Reference-based annotation methods are also limited by the availability of cell references and the small number of cells in the reference datasets. By contrast, scFMs offer a new paradigm by leveraging learned cell representations to classify cell types in an unbiased, data-driven manner. Because scFMs have been exposed to countless cell states during pretraining, they can embed cells in a latent space where similar cells would be embedded, even without predefined marker genes. In practice, a foundation model can generate an embedding for a new cell and use a classifier to predict the cell type. These cell embeddings can be treated as universal cell embeddings that share latent spaces with millions of cells in pretrained datasets. In fact, foundation models have achieved state-of-the-art performance on cell-type-labeling benchmarks with various datasets^2,42^. Moreover, scFMs can be fine-tuned on a small set of labeled cells to further improve their accuracy on a specific tissue or experimental platform. Incorporating datasets that capture a broader spectrum of cellular states such as those involving developmental trajectories, perturbations or transcription factor atlases could potentially enhance cell-type-annotation performance by enriching the model’s exposure to varied transcriptional programs^25^. An important note is that scFMs, when used in a zero-shot, may sometimes misclassify rare or novel cell types if they were not well-represented in the training data^24,25^. Nonetheless, as the size, diversity and quality of pretraining datasets grow, the expectation is that foundation models will increasingly cover the full spectrum of cell identities and states, making them powerful annotators for single-cell data with much less human bias.

### Batch correction and data integration

A key advantage of single-cell analysis is its ability to integrate numerous publicly available datasets from disparate experiments or batches. However, the inherent complexity and challenges of such integration render the task difficult^44^. Because scFMs are trained on extremely diverse data, they learn to focus on biological variation while ignoring technical noise. The cell embeddings produced by a good foundation model should, in theory, place biologically similar cells together even if they come from different batches. Indeed, scFMs have proven to be useful for batch correction and data integration by embedding cells from multiple datasets into a common latent space. Whereas some foundation models function without explicit batch normalization steps, the other models explicitly include a training objective to discourage encoding batch-specific signals^7^. In addition, scFMs are being explored for multiomics integration. A foundation model can be pretrained and fine-tuned on transcriptomes and/or epigenome data to project both modalities into one space. Early results indicate that such models can align single-cell data across modalities, enabling joint analyses^12^. In summary, by learning a representation that emphasizes true biological signals over technical artifacts, scFMs act as robust tools for single-cell data integration across batches, modalities and even species.

### Imputation

scRNA-seq data are inherently sparse but also contain false zeros due to dropout events where a gene is expressed but undetected^45^. scFMs, with their learned gene–gene and cell–cell relationships, provide a sophisticated method for the imputation of missing values. In theory, foundation models can deduce the expected gene expression in each cell on the basis of its contextual relationships. Concretely, an scFM can take a cell’s partially observed gene expression profile and predict the values of genes that are missing or underexpressed. The attention mechanisms and deep neural layers allow the model to capture nonlinear dependencies, allowing it to leverage global knowledge learned from numerous cell-to-cell and gene-to-gene relationships. This enables the scFM to make informed guesses about how gene profiles are generally distributed in a certain cell or what genes are part of a common module. Although imputation is possible with most scFMs, user-friendly built-in functionality is often not available, limiting their routine application for downstream analyses. Furthermore, there is a report of low performance for single-cell expression imputation, though results may be better for spatial transcriptomics data^2^. However, several models have started to incorporate imputation into the modeling itself, suggesting that performance could improve with more targeted or advanced approaches^19^. An effective imputation not only restores the true biological signal obscured by technical noise but also enhances substantially the accuracy and interpretability of key downstream analyses, especially gene-related tasks. Nevertheless, careful evaluation is necessary to assess the risk of generating inaccurate values, particularly for underrepresented cell types. Therefore, fine-tuning the models on specific datasets can improve the imputation accuracy in these cases. Overall, scFMs provide a context-aware way to address sparsity, helping to denoise single-cell data and reveal signals that would otherwise be lost due to technical dropouts.

### Gene function prediction

Functions of genes can be highly context-dependent, varying between cell types, developmental stages or abnormal conditions. Analysis of gene functions from a single cell or a group of similar cells is a key component in answering biological questions with single-cell datasets. scFMs can learn latent representations for genes that encode aspects of gene function and regulation. The idea is analogous to how language models learn word embeddings that capture semantic meaning. Extracted from these models, genes with similar expression patterns across many cells and conditions will have similar embeddings. These embeddings can be used to predict gene functions. If an uncharacterized gene has an embedding close to that of a well-studied gene or if groups of genes are within proximity, they may share functional roles or participate in the same pathway. By analyzing these learned representations, researchers can identify novel functions of genes in different contexts and even predict new candidate disease genes. Another approach is to use the model to fine-tune with perturbation datasets or perform in silico perturbations to observe which genes react, thereby inferring the function. The attention mechanisms in these models allow the identification of much more complex patterns to define interactions among genes. As these models are refined, we expect their gene embeddings to be increasingly used for tasks such as predicting transcription factor targets, annotating single-cell-specific gene sets or prioritizing genes in genome-wide association studies by linking them to cellular phenotypes.

### Gene network inference

A gene network is a structured representation of the relationships among genes connected through coexpression, regulatory interactions or functional associations. Deriving regulatory or functional gene networks from single-cell data is a complex task, as traditional correlation analyses often result in unreliable interactions or fail to distinguish direct and indirect functional connections^46^. scFMs approach this problem by leveraging their attention weights and gene embeddings to infer gene networks. In a transformer, each attention head can be thought of as learning a specific kind of relationship between tokens^32^. In the context of scFMs, certain attention heads may focus on regulatory links or functional connections. By extracting and analyzing the attention matrices from a trained model, a putative gene network can be constructed. Beyond attention, the distance between gene embeddings or their modular structures can also indicate network connections. Fine-tuning a foundation model on a specific network inference task can further sharpen its ability to predict edges accurately. Network generation with scFMs can provide a versatile framework through which both context-agnostic networks and context-specific networks can be inferred. The original attention weights or embeddings from the whole model provide a general network that persists across many cell states, whereas embeddings extracted with condition- or cell-type-specific data subsets can output context-specific networks. These network inferences from scFMs are valuable for generating new hypotheses about gene interactions and gene modules in certain biological conditions or diseases.

### Gene perturbation

A particularly exciting application of scFMs is their use in simulating cellular responses to perturbations, functioning as “virtual cells”^47^. Traditionally, such responses would require actual perturbation experiments, but scFM can simulate these effects in silico in a genome-wide manner. During pretraining or fine-tuning, a model can be exposed to perturbation datasets with CRISPR screening or drug treatment so that it learns to associate specific interventions with specific transcriptional changes. Once trained, the model’s latent space can be traversed to generate a predicted postperturbation gene expression profile for a given initial cell state^7^. Even without additional datasets, the values or ranks of certain genes in the models could be altered to mimic certain perturbations such as knockout or overexpression^14^. By analyzing changes in the latent spaces of scFM before and after perturbation, researchers can identify which genes or cells are more likely to be affected. This method also enables prediction about how this perturbation would influence cellular phenotypes. The ability to predict the outcomes of perturbations can guide experimental design by highlighting the most impactful genes to perturb or suggesting which drug might drive a desired cell state change. Although they are not perfect, these models provide a scalable and hypothesis-generating tool for perturbation biology. Furthermore, there is a potential for improvement as more perturbation data become available for training and as better methodologies for in scFM-based in silico perturbation are developed.

### Empowering experimental biology with scFMs

With the downstream tasks listed above, scFMs can be incorporated to generate novel hypotheses and to guide experimental design, ultimately leading to new biological insights. For instance, scGPT demonstrates considerable potential in uncovering new biology through advanced perturbation analysis. It not only predicts the transcriptional consequences of specific genetic perturbations but also performs the reverse—identifying potential genetic interventions that can achieve a desired cellular state^7^. This bidirectional capability allows researchers to generate novel hypotheses about the underlying drivers of cell identity or disease and to prioritize perturbation targets that are likely to produce desired phenotypic outcomes.

SCimilarity focuses on learning cell representations. By leveraging these representations, the model enables the matching of in vivo cell states with similar in vitro cell states from publicly available datasets^38^. This capability can help researchers design experiments to test or model specific cell states of interest.

Geneformer further illustrates how scFMs can facilitate biological discovery, especially when adapted to specific domain contexts. Fine-tuned on large-scale cancer datasets, it has been used to predict clinically relevant features such as mismatch repair status and cell type annotations within the tumor microenvironment. Moreover, its in silico perturbation analyses have identified novel candidate genes that may drive malignant cells into less harmful states or induce activated T cell states with potential therapeutic benefit^18^.

These predictive capabilities are crucial for identifying therapeutic targets and generating testable hypotheses that advance our understanding and treatment of diseases such as cancer. Collectively, these examples illustrate how scFMs can serve as powerful computational tools for experimental biologists to interrogate complex biological systems, model disease processes and accelerate therapeutic discovery.

## Recent developments in scFMs

**Fig. 3:**
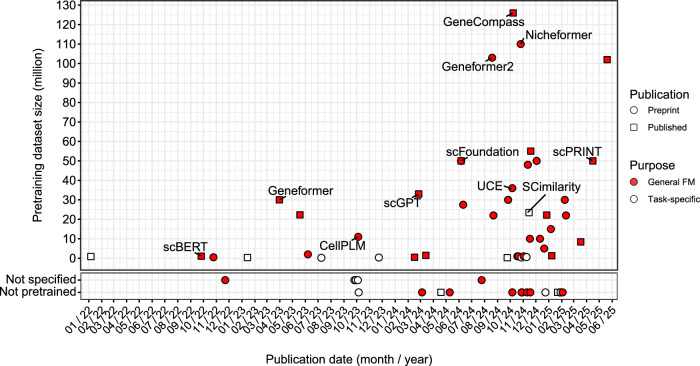
Summary of recently developed scFMs. Various scFMs and task-specific models utilizing scFMs. The shape of each marker indicates its publication status as of May 2025 (for example, preprint or peer-reviewed scientific journal), whereas the color coding distinguishes the tool’s purpose and characteristics, categorizing them as either general scFMs or task-specific models that leverage scFMs. The scFMs mentioned in Table 1 are labeled with their model names.

Since 2022, numerous models for single-cell analysis have emerged that either generate general-purpose scFMs with multiple downstream tasks or incorporate scFMs within frameworks for specific tasks. Although it remains challenging to evaluate and compare all these models partly because many are still available only as preprints, we observe a clear trend of an increase in the sizes of pretraining datasets alongside the emergence of task-specific approaches that integrate existing scFMs or other foundation models (Fig. 3). For each model, we provide a brief summary of its publication status, the types and sizes of omics data used for pretraining and a simplified overview of its model architecture (Supplementary Table 1). Moreover, we highlight several noteworthy models—scBERT^13^, geneformer^14,18^, tGPT^29^, CellLM^15^, CellPLM^48^, scGPT^7,10^, scFoundation^34^, scPRINT^19^, UCE^16^, GeneCompass^17^, Nicheformer^11^ and SCimilarity^38^. For these selected models, we also summarize the sources of the pretraining datasets, input modification methods, pretraining tasks and downstream tasks available either as built-in functions or through tutorials (Table 1).

**Table 1:** Summary of selected scFMs.

Model name	Journal	Date	Omics	Dataset size	Pretraining dataset	Architecture	Input modification	Pretraining tasks	Downstream tasks*
scBERT	*Nature Machine Intelligence*	September 2022	scRNA-seq	1.1 million	PanglaoDB. 209 human datasets and 74 tissues	Encoder-only (performer)	Value binning	Gene masking	Cell type prediction and novel cell type detection
Geneformer	*Nature*	May 2023	scRNA-seq	30 million	Human Cell Atlas, EMBL-EBI Single Cell Expression Atlas, PanglaoDB, Tumor-Immune Single-Cell Hub, GEO and SRA. Human only, no malignant cells or immortalized cell lines	Encoder-only	Ranked by expression and normalize gene expression by median value from pretraining dataset	Gene masking	Cell type prediction, in silico perturbation, gene classification and cell/gene embedding
tGPT	*iScience*	May 2023	scRNA-seq	22.3 million	Human Cell Atlas, Single Cell Expression Atlas, COVID-19 Atlas, Tabula Muris and Mouse Cell Atlas	Decoder-only	Ranked by expression	Autoregressive gene prediction	Cell embedding
CellLM	arXiv	June 2023	scRNA-seq	2 million	PanglaoDB and CancerSCEM	Encoder-only (performer)	Value binning and PPI embedding	Gene masking and contrastive learning (cell)	Cell type prediction
CellPLM	bioRxiv	October 2023	scRNA-seq and spatial	11 million	Human Tumor Cell Atlas, Human Cell Atlas, GEO and CosMx dataset	Encoder	Value projection	Gene masking	Cell type prediction, spatial gene imputation and cell embedding
scGPT	*Nature Methods*	February 2024	scRNA-seq, scATAC-seq and CITE-seq	33 million	CELLxGENE, Human Cell Atlas and PanglaoDB	Decoder-inspired with masked generative pretraining	Value binning	Attention masking	Cell type prediction, in silico perturbation, batch integration, multiomics integration, gene regulatory network generation and reference mapping
scFoundation	*Nature Methods*	June 2024	scRNA-seq	50 million	GEO, Human Cell Atlas, EMBL-EBI, hECA and DISCO	Asymmetric encoder–decoder	Value projection	Gene masking	Cell type prediction, perturbation prediction (GEARS), cell/gene embedding, drug response prediction (DeepCDR), read depth enhancement, gene module and network inference
Geneformer2	bioRxiv	August 2024	scRNA-seq	103 million	Geneformer1 + Broad Institute Single Cell Portal, CELLxGENE and Brotman Baty Institute-Allen Single Cell Atlases	Encoder-only	Ranked by expression and normalize gene expression by median value from pretraining dataset	Gene masking	Cell type prediction, in silico perturbation, gene classification, cell/gene embedding and multitask fine-tuning
UCE	bioRxiv	October 2024	scRNA-seq	36 million	CELLxGENE. Eight species	Encoder	ESM2-based embedding and ordered by genomic location	Gene masking and binary classification of expression	Cell embedding
GeneCompass	*Cell Research*	October 2024	scRNA-seq	126 million	GEO, ArrayExpress, China National Center for Bioinformation and CellxGENE. Human and mouse	Encoder-only	Ranked by expression and embeddings from prior knowledge	Gene masking for gene ID and expression prediction	Cell type prediction, perturbation prediction (GEARS), GRN inference, drug response prediction and gene embedding
Nicheformer	bioRxiv	October 2024	scRNA-seq and spatial	110 million	GEO, CosMx, Xenium and MERFISH. Human and mouse	Encoder-only	Ranked by expression	Gene masking for gene rank prediction	Cell embedding and niche prediction
SCimilarity	*Nature*	November 2024	scRNA-seq	23.4 million	GEO, CELLxGENE and manual curation	Non-transformer encoder–decoder model	Value projection	Cell similarity and expression reconstruction	Cell type prediction and cell search
scPRINT	*Nature Communications*	April 2025	scRNA-seq and scATAC-seq	54 million	CELLxGENE. Human and mouse	Encoder–decoder	Normalized expression, ESM2-based embedding and gene location as position	Denoising, label prediction and expression reconstruction	Cell type prediction, GRN inference and cell embedding

^*^Tasks available as built-in functions or tutorials. *CITE-seq* Cellular Indexing of Transcriptomes and Epitopes by Sequencing), *GRN* gene regulatory network, *PPI* protein-protein interaction.

## Current limitations and challenges

Although scFMs are powerful new frameworks, they come with several limitations and challenges. In the following two sections, we discuss current issues in model architecture, usages and performances and outline future directions to address these challenges and extend the capabilities of scFMs.

### Pretraining challenges: data representation and quality

#### Tokenization and independence assumptions

Nearly all scFMs treat each cell independently as a separate training example analogous to sentences in a text corpus. This ignores the potential relationships among cells, such as cellular interactions, signaling or spatial organization. The current approach may be suboptimal for analyzing cellular environments with higher intercellular signaling or spatially resolved transcriptomics where neighboring cells influence each other. Future models might introduce two-way attention^6^ for both cells and genes or graph-based modules to account for cell–cell relationships during pretraining, allowing the model to learn cell interaction networks. In addition, the methods used to impose an order on genes might not be fully optimized in biological context. Ranking or binning genes by expression provides a deterministic sequence, but this may not fully capture the true biological signal from cells. Genes do not have a natural linear order; therefore, any sequential modeling is an approximation. Exploring alternative methods to better represent biological connections or models optimized for tabular structures could improve how well the model learns from cellular or gene–gene interactions.

#### Data quality and heterogeneity

The massive datasets used for pretraining scFMs are a double-edged sword. On the one hand, more data provide broader coverage; on the other hand, not all data are equally reliable. Cells compiled from many studies can introduce extensive variability due to differing sampling and sequencing protocols. Moreover, single-cell data inherently contain artifacts such as doublets, ambient RNA contamination and other noises^49^. If these artifacts are prevalent in the training data, the model may learn spurious patterns. Removing the low-quality cells and filtering technical artifacts are essential before using them to train an scFM. However, doing this at the scale of tens of millions of cells is challenging^50^. Automated data curation pipelines and rigorous quality control metrics need to be integrated into the model training workflow. As scFMs move forward, there is a need for better data preprocessing and curation at scale, potentially leveraging community efforts to standardize single-cell data.

#### Redundancy and dataset overlap

When aggregating data from public resources, a hidden issue is overlapping datasets. The same cells or very similar cells might appear in multiple sources. If not addressed, the model could effectively see the same cell multiple times, giving it an outsized influence, and could result in data leakage for situations where the separation of training and test datasets are necessary. One approach is to use the hashing of cell transcriptomes or metadata to identify probable duplicates and remove them. For example, in the development of an updated Geneformer model, a deduplication strategy was performed to ensure that no cell was counted twice^18^. Such strategies will probably become standard as foundation model training pipelines mature.

#### Quantity versus quality of data

Empirical evidence from foundational models across various fields, combined with intuitive reasoning, suggests that increasing data volume typically enhances model performance. However, recent observations suggest that more datasets might not always be better^24^. If additional data are noisy or not representative of new biology, they might just add noise to the training process. These results indicate that beyond a certain point, increasing the number of cells or studies in the training set yields diminishing returns on downstream performance. In several cases, focusing on the diversity of data to cover various biological phenomena and cellular states is more important than the sheer number of cells. This suggests that a curated dataset for pretraining could outperform a naively aggregated superset of all available data. It may even be beneficial to exclude some data that could overrepresent or misrepresent certain cellular populations. For future scFMs, research could focus on data selection techniques to decide which cells to train to optimize generalization. This intersects with continual learning and fine-tuning as well—adding data in stages and evaluating how they improve model performance on a validation set of tasks could inform whether adding another million cells from a given new study is useful or not.

### Model usage and deployment

#### Rare cell types and class imbalance

Single-cell datasets often have highly imbalanced cell type distributions. Foundation models trained on such data will inherently be biased toward the predominant cell types, potentially undermining performance on rare cell types. Recent studies examined how scFMs handle imbalanced data and found that models struggled to reliably annotate minority cell populations in a zero-shot setting^2,24^. Possible remedies involve data augmentation or reweighting during training by oversampling rare cell types or adjusting ratios of cell types or datasets from different conditions. Another approach is to fine-tune the foundation model on a dataset that is balanced or specifically enriched for certain cell types that better represent conditions of interest. This can recalibrate the model’s internal representations to give more attention to features relevant to those cells. An encouraging aspect is that foundation models can even learn cellular patterns if given experimental datasets designed to represent intercellular variability or progression^25^. This suggests potential in augmenting scFMs with carefully designed datasets that capture certain developmental or phenotypical phenomena. In practical applications, users of scFMs should be cautious when applying a model to datasets with rare cell types or condition-specific populations. For the modeling of scFMs, careful considerations for both the quantity and quality of pretraining datasets are necessary.

#### Gene embedding interpretability and usage

When utilized for downstream tasks, gene embeddings are often obtained by averaging the extracted embeddings across groups of cells similar to pseudobulk approaches used for single-cell analyses^14^. However, because not every cell expresses every gene, and because often only the top-expressed genes per cell are used as tokens, there is an inherent bias in how gene embeddings are derived. If embeddings are simply averaged across all cells, genes that are not broadly expressed will appear more generic than they truly are, especially if these embeddings are extracted from heterogeneous populations. In other words, the heterogeneity of expression can lead to embeddings that may mix multiple contexts or lose specificity. There are still limited evaluations on the credibility of these embeddings, and future work should consider how to successfully extract and utilize the gene embeddings through methods specific to scFMs. Another idea is to include a gene-centric pretraining or fine-tuning objective as part of the multitask training. This could force gene embeddings to align better with the data context.

#### Attention weights and interpretability

One of the major characteristics of transformer models is attention mechanisms, which can learn connections among input features. However, previous studies have indicated that attention is not always a direct indicator of the feature importance or interaction^51^. In scFMs, attention weights are often utilized similar to the gene embeddings to identify interactions and connections among genes. However, similar to NLP studies, certain attention heads might not capture biologically relevant gene connections. Thus, treating attention matrices as biological networks should be done with caution. Until then, any networks or feature attributions derived from attention matrices should ideally be cross-checked against other methods or known biology.

### Benchmarking and evaluation

Evaluating scFMs presents a unique challenge. Unlike in NLP, where there are standard benchmarks and community efforts that cover a range of tasks, the single-cell field lacks a unified benchmarking framework. Each scFM is often introduced with its own set of downstream tasks and evaluations, which makes it difficult to compare models head-to-head. As a result, there are only a few benchmark papers, with most of them covering limited models. In addition, what constitutes success for a foundation model in biology is broad, and success for certain downstream tasks does not necessarily indicate a good biological model.

To address this, the community is beginning to propose benchmarking frameworks. One example is the scEval pipeline, which evaluates foundation models across eight canonical single-cell tasks under consistent conditions^2^. In their evaluation, they not only compared task performance but also considered user accessibility and training stability. Similar to this study, establishing a common set of benchmark datasets for pretraining and a common suite of downstream test sets would greatly facilitate progress. Beyond datasets and downstream tasks, other important aspects of foundation models such as zero-shot utility, fine-tuning performances and usage efficiency (memory and time) should be considered. In summary, improving benchmarking will shine light on where scFMs truly add value and where they need improvement, ultimately guiding the development of the next-generation models.

### Performance limitations and scope

#### Zero-shot performance versus task-specific models

A recurring observation is that scFMs in zero-shot mode often do not outperform existing tools that were specifically designed for a specific task^2^. Fine-tuning could narrow this gap, but fine-tuning itself is a nontrivial process, as it requires computational resources, careful hyperparameter selection, enough representative data and considerable knowledge for modeling. However, scFMs can still provide comprehensive analysis frameworks that can provide capacities for multiple tasks with moderate-to-good performance and consistency that is less reliant on users and less susceptible to dataset-specific effects. Furthermore, by directly and indirectly incorporating information from large bodies of single-cell datasets, outputs could be more interpretable by providing relative information in comparison with other datasets. Over time, as scFMs improve, there is more potential to provide state-of-the-art performance for multiple tasks and capacities to be utilized for tasks that are not currently performed by specialized tools.

#### Practicality of fine-tuning and deployment

Fine-tuning scFM can be resource-intensive and require extensive knowledge when built-in methods or tutorials are not sufficient. This raises an issue of accessibility for many labs and biologists. For limitations in computational resources, lightweight versions could be modeled as in the field of NLP. Fine-tuning can also be made more user-friendly by providing tutorials for various tasks or scenarios that might require fine-tuning. As these models become more common as in NLP, the scFM ecosystem will probably improve to include tools and frameworks more accessible to nonexperts, promoting broader usage and innovation in the field.

## Future directions

### Novel applications of scFMs

Beyond enhancing existing downstream tasks, future research should explore novel applications uniquely enabled by scFMs. By leveraging large pretraining datasets and capturing a wide spectrum of biological phenomena, scFMs can accurately model the complex distributions inherent in single-cell data. This capability paves the way for innovative tasks such as synthetic cell or dataset generation. When these models are pretrained or fine-tuned with metadata that encode specific cellular conditions or states, they can generate synthetic cells reflecting desired biological contexts. Such tailored synthetic data not only enable rigorous testing of algorithmic efficacy and resilience under diverse conditions but also facilitate the exploration and validation of intricate biological hypotheses.

In addition, in silico perturbation stands out as a promising application area for scFMs. With many models already incorporating perturbation analysis, researchers can simulate targeted interventions and observe resultant changes in gene expression and cellular behavior. This approach provides invaluable insights into gene functions and cellular dynamics, thereby advancing our understanding of complex biological systems. Expanding these tasks, the ultimate goal would be to generate virtual cells that faithfully recapitulate the behaviors of their real-world counterparts^47^. For this goal, models that incorporate multiomics profiles and high performances for mentioned downstream tasks would be necessary. Beyond these tasks, many unexplored applications remain that could further harness their unique capabilities. Researchers are encouraged to focus on these emerging directions, as they hold the promise of unlocking new insights into cellular behavior and transforming biological research.

### Multiomics and epigenomic integration

Current models are predominantly exploring scRNA-seq datasets owing to their availability and ease of interpretation. However, to capture the full complexity of cellular identity, scFMs must extend their scope beyond transcriptomes. By incorporating additional omics layers such as epigenetic features including DNA methylation and histone modifications^52^, chromatin accessibility from scATAC-seq^53^, spatial context^54^ and protein expression^55^, models can better elucidate intricate relationships among gene expression, regulatory genomic elements, cellular interactions and translated phenotypes. Models such as Nicheformer and scGPT-spatial are attempting to incorporate multiple omics datasets^10,11^. Although these additional dimensions will necessitate more complex tokenization strategies and larger model architecture, they promise to deliver a more holistic representation of the cell. Moreover, leveraging joint sequencing (multiomics sequencing) datasets can further bridge the gaps between various omics profiles, enabling more comprehensive and integrative analyses^56^.

### Cross-species models

Biology often benefits from comparative analysis across species. Furthermore, datasets derived from model organisms provide an invaluable opportunity to analyze experimentally engineered conditions that are seldom observed in typical human samples, thereby offering unique insights into cellular behavior under diverse scenarios. A future scFM might be trained simultaneously on data from human, mouse and other model organisms, learning a species-agnostic representation of cell states. If done successfully, this could enable transferring knowledge from well-studied systems to human biology by aligning cell embeddings across species. There are early attempts, such as the GeneCompass model that uses knowledge-informed training across species^17^. Ultimately, by bridging the biological connections between model organisms and humans, these cross-species models hold the potential to revolutionize our understanding of disease mechanisms and guide the development of more effective therapies.

### Model efficiency and accessibility

Improving the efficiency and accessibility of scFMs is important. Building on trends in large language models, future research should consider model compression techniques such as quantization, distillation and pruning to reduce computational demands^57^. Furthermore, developing dedicated scFM platforms with online resources for biologists and laboratories without GPU capabilities would substantially broaden the accessibility. For usability, models should provide more user-friendly tutorials and built-in functions that support a range of downstream tasks and model-tuning operations.

### Ethical and interpretative considerations

With growing concerns about data privacy and the ethical implications of increasingly powerful models, it is imperative for scFMs to integrate robust privacy preservation measures and reduce ethical risks^58^. As these models become larger and more capable, ensuring their responsible use is critical. For example, a recent study featuring the Tabula model demonstrated the potential of privacy-preserving techniques using federated learning, which decentralizes the training process and avoids sharing sensitive biological data^59^. By adopting such approaches, researchers can ensure that advancements in scFMs are both ethically responsible and aligned with stringent privacy standards.

## Discussion and conclusion

scFMs represent a convergence of big data biology and state-of-the-art AI. They have demonstrated the ability to learn from massive single-cell datasets and perform a spectrum of tasks, from labeling cells and imputing missing values to predicting experimental perturbations and uncovering gene networks. In essence, an scFM serves as a comprehensive analytical engine, potentially replacing dozens of separate tools with one model that can be applied in different modes. This review has highlighted that although current scFMs such as Geneformer, scGPT and scFoundation are already pushing the boundaries, there remain important limitations in need of attention. Data quality, model interpretability and evaluation standards are key areas to improve so that these models can be reliably used by the wider biomedical community.

It is also evident that scFMs are still in their early days. The field is rapidly evolving, and each new iteration of a foundation model raises the bar with improved design or broader data integration. Even task-specific tools are beginning to partially integrate scFMs into their pipelines. With continued interdisciplinary collaboration, we can expect future models to become even more powerful and versatile by encompassing multiomics profiles and other biological factors all within one framework. If successful, such models could answer complex questions that would otherwise require piecing together information from separate studies or datasets.

Crucially, scFMs should be viewed as a complement to and not a replacement for expert-driven analysis. They are tools that can find patterns in data at a scale and complexity that humans cannot easily parse, but the interpretation of those patterns and the design of validation experiments remain in the realm of biological expertise. Going forward, a tight coupling among model predictions, additional computational analysis and experimental testing will be essential. As more biologists start to use the foundation models, feedback from real-world use will inform better models for continuous improvement.

In conclusion, scFMs offer a promising foundation for the next generation of computational biology. By embedding the knowledge from vast single-cell compendia, they enable researchers to extract meaningful information from new data with unprecedented ease and depth. There are clear challenges to overcome, but the trajectory of recent research suggests that many of these will be surmounted. In the coming years, we anticipate scFMs will become increasingly standard in single-cell analysis pipelines. Their ability to unify diverse analyses can streamline research workflows and perhaps lead to discoveries that were previously hidden in the complexity of high-dimensional data. The integration of single-cell genomics and AI as a foundation model thus stands as an exciting frontier, holding great potential to accelerate our understanding of cells in health and disease and to drive forward the era of data-driven biology.

## Supplementary information

Supplementary Table 1
